# Rapid CT-based Estimation of Articular Cartilage Biomechanics in the Knee Joint Without Cartilage Segmentation

**DOI:** 10.1007/s10439-020-02666-y

**Published:** 2020-11-11

**Authors:** Ali Mohammadi, Katariina A. H. Myller, Petri Tanska, Jukka Hirvasniemi, Simo Saarakkala, Juha Töyräs, Rami K. Korhonen, Mika E. Mononen

**Affiliations:** 1 Department of Applied Physics, University of Eastern Finland, POB 1627, 70211 Kuopio, Finland; 2 Diagnostic Imaging Center, Kuopio University Hospital, Kuopio, Finland; 3 Department of Medical Physics, Turku University Central Hospital, 20500 Turku, Finland; 4 Department of Radiology & Nuclear Medicine, Erasmus University Medical Center, Rotterdam, The Netherlands; 5 Research Unit of Medical Imaging, Physics and Technology, Faculty of Medicine, University of Oulu, Oulu, Finland; 6 Department of Diagnostic Radiology, Oulu University Hospital, Oulu, Finland; 7 School of Information Technology and Electrical Engineering, The University of Queensland, Brisbane, Australia

**Keywords:** Finite element (FE) modeling, Articular cartilage, Atlas-based modeling, Computed tomography, Magnetic resonance imaging, Osteoarthritis (OA)

## Abstract

Knee osteoarthritis (OA) is a painful joint disease, causing disabilities in daily activities. However, there is no known cure for OA, and the best treatment strategy might be prevention. Finite element (FE) modeling has demonstrated potential for evaluating personalized risks for the progression of OA. Current FE modeling approaches use primarily magnetic resonance imaging (MRI) to construct personalized knee joint models. However, MRI is expensive and has lower resolution than computed tomography (CT). In this study, we extend a previously presented atlas-based FE modeling framework for automatic model generation and simulation of knee joint tissue responses using contrast agent-free CT. In this method, based on certain anatomical dimensions measured from bone surfaces, an optimal template is selected and scaled to generate a personalized FE model. We compared the simulated tissue responses of the CT-based models with those of the MRI-based models. We show that the CT-based models are capable of producing similar tensile stresses, fibril strains, and fluid pressures of knee joint cartilage compared to those of the MRI-based models. This study provides a new methodology for the analysis of knee joint and cartilage mechanics based on measurement of bone dimensions from native CT scans.

**Electronic supplementary material:**

The online version of this article (10.1007/s10439-020-02666-y) contains supplementary material, which is available to authorized users.

## Introduction

Osteoarthritis (OA) is the most common arthritic disease and is the leading cause of disability in the United States and other developed countries.^31^,^48^ Knee OA, which prevalence has doubled since the mid-20^th^ century, is the most prevalent form of OA.^49^ However, there is no cure for knee OA, and therefore, the best and the most cost-effective treatment option might be prevention. Although substantial evidence indicates that mechanical loading and local tissue stresses and deformations are one of the main driving factors for knee OA,^5^,^13^ direct measurement of the knee joint stresses and strains *in vivo* is impractical and ethically very questionable. Over the past two decades, computational finite element (FE) models have made remarkable advances in enabling a quantitative estimation of the local tissue stresses and deformations applied to the soft tissues of the knee joint during different loading conditions.^1^,^2^,^9^,^11^,^15^,^28^,^38^,^50^ These biomechanical parameters have been utilized in predictive FE models to simulate personalized risks for the onset and progression of knee OA.^30^,^35^,^47^ Nevertheless, several obstacles need to be overcome prior to clinical use. These obstacles include the long time and high technical expertise required for generating FE models via manual image segmentation and meshing.

Magnetic resonance imaging (MRI) and computed tomography (CT) are the most common imaging modalities used to acquire geometries for computational FE models of the knee and hip joints.^2^,^4^,^6^,^9^,^17^,^28^,^47^ MRI enables visualizing soft tissues and even analysis of cartilage integrity and deformation.^26^ Therefore, MRI is typically used to obtain knee joint geometries for FE models. However, MRI suffers from relatively long acquisition time and low signal-to-noise ratio when high spatial resolution is required. Moreover, it may be unsuitable for patients with implants, pacemakers, or claustrophobia. As an alternative to MRI, a contrast-enhanced CT (CECT) imaging method has been used for generating knee and hip joint FE models.^4^,^6^,^17^,^33^,^47^ Compared to MRI, CECT has superior resolution and contrast, and enables better characterization of cartilage lesions.^32^ However, this method has not yet been extensively applied clinically. Native CT, similar to CECT, benefits from very short acquisition time, enables quantitative assessment of the subchondral bone, is cost-effective, and requires no contrast agent injection before imaging. A limitation with this method is that cartilage thicknesses have to be assumed or obtained from MRI.^34^ Therefore, it would be highly beneficial to develop a method for generation of knee joint FE models with personalized cartilage volumes and topographies based on native CT images.

Various methods have been developed for fast and easy FE knee joint model generation and simulation. Semiautomatic and automatic segmentation methods have been developed to generate personalized FE models.^14^,^32^,^37^,^44^,^53^ However, due to the image and segmentation quality, the required manual effort for the FE model generation and simulation, e.g., meshing the volumes and running the models properly, remain challenging. Rodriguez-Vila et al.^42^ introduced a new promising approach for the rapid and automatic generation of FE meshes. Nevertheless, they did not test their approach with a large group of subjects. Importantly, even if segmentation and meshing would be fully automated, yet, the user has to assign the material properties and implement the boundary conditions in the model. Furthermore, most of the developed methods can only be used with MRI or CECT,^14^,^32^,^37^,^42^,^44^,^53^ and there are no methods for rapid generation of personalized FE models of the knee joint from contrast agent-free CT.

To address this, our objective is to expand a previously developed atlas-based knee joint FE modeling framework^29^ to generate and simulate knee joint models based on contrast agent-free CT with minimal manual intervention. With this framework, one can automatically construct knee joint FE models with the material properties of the tissues and loading conditions. To verify our framework, we (1) compare the anatomical dimensions (needed to generate FE models automatically) measured from contrast agent-free CT and MRI, (2) estimate the intrarater reliability of the presented atlas-based method for both imaging modalities, and (3) compare the results of the atlas-based FE models generated from contrast agent-free CT and MRI modalities.

## Materials and Methods

**Figure 1:**
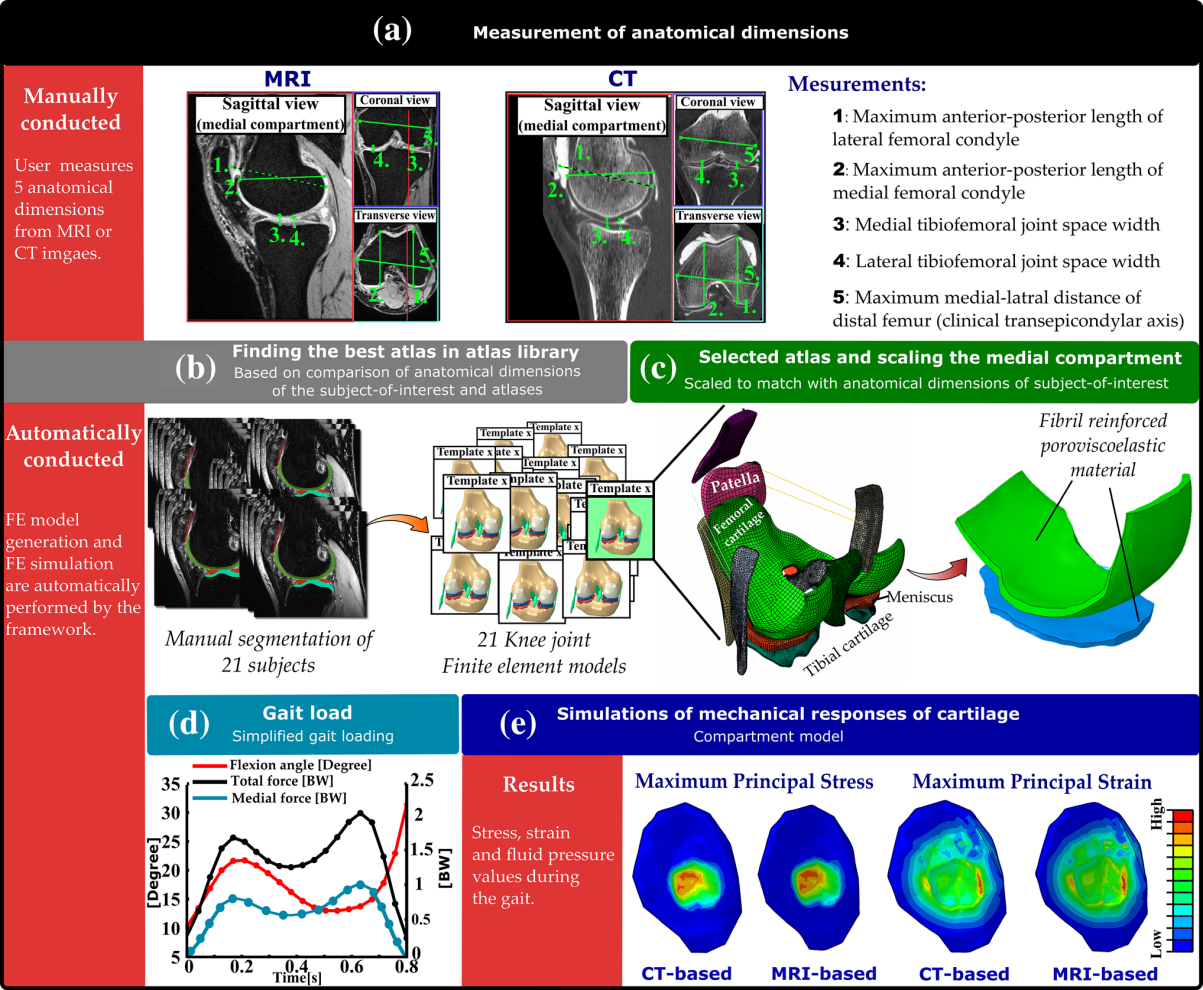
The workflow of the study. An illustration of the atlas-based framework for the FE modeling of the knee joint. The first row (part a) demonstrates the manual work required for model generation and simulation. For each subject, five shape parameters (anatomical dimensions of the femur) are measured by the user (a). By comparing these values with the same anatomical dimension values in 21 atlases (b), the most suitable atlas is selected (c). This atlas includes assigned material properties and meshes, which have been previously validated. Next, the medial compartment is scaled in medial-lateral, anterior-posterior and thickness directions with respect to the anatomical dimensions of the subject-of-interest (c). The scaled template model is simulated using the simplified gait loading (d). Finally, by simulating the model in Abaqus, the biomechanical outputs of the knee joint are obtained (e).

The workflow of the utilized atlas-based method is shown in Fig. 1.

### Subjects and Image-Acquisition

Nine patients (two males and seven females aged between 50 and 68 years) were enrolled in the study after they had provided written consent.^18^ The Ethical Committee of the Northern Ostrobothnia Hospital District approved the study (decision No. 33/2010). We acquired CT images using a clinical CT-scanner (Discovery, PET/CT, 690 GE Medical Systems, Waukesha, WI, USA) with a tube voltage of 100 kV, pitch of 0.53, voxel size of 0.3 × 0.3 × 0.3 mm^3^, and 4.3 mm of aluminum as the filter. We acquired knee joint MR images using a 3 T scanner (Siemens Skyra, Siemens Healthcare, Erlangen, Germany) with a 15-channel transmit/receive knee coil (Quality Electrodynamics (QED), Mayfield Village, OH, USA). The MRI acquisition sequence was double echo steady-state (DESS) with water excitation and the parameters as follows: repetition time = 14.1 ms, echo time = 5 ms, field-of-view = 150 × 150 mm^2^, matrix size = 256 × 256, slice thickness = 0.6 mm, and voxel size = 0.6 × 0.6 × 0.6 mm^3^.

### Template Approach

In order to generate FE models, we utilized the same approach as originally developed in our previous study for MR images.^29^ First, the anatomical dimensions of the distal femur and tibiofemoral joint space width were measured from MR and CT images (Fig. 1a). The measured dimensions included tibiofemoral joint space width from both medial and lateral compartments (JSW medial and JSW lateral), maximum anterior-posterior dimensions from medial and lateral condyles considering the orientation of condyles (AP medial and AP lateral), and maximum medial-lateral dimension determined from the maximum distance between medial and lateral epicondyles, *i.e.*, the clinical transepicondylar axis (ML).

Thereafter, the anatomical dimensions were normalized to the measured ML value to allow comparison between the models in terms of shape.^21^,^29^,^43^ Subsequently, the normalized dimensions of the subject-of-interest were compared to the normalized dimensions of 21 FE models in the atlas library, taking less than 10 seconds of computational time (3.4 GHz Intel Core i7 processor, 16 GB RAM computer) (Fig. 1b). The most suitable atlas was selected based on the minimum root mean square error (RMSE) in the normalized anatomical dimensions between the subject-of-interest and atlas library (Figs. 1b and 1c). Finally, the selected atlas was scaled to match the anatomical dimensions of the subject-of-interest in the Cartesian coordinate system (Fig. 1c). In the scaling procedure, the anterior-posterior, medial-lateral, and tibiofemoral joint space width directions corresponded to the respective Cartesian axes x, y and z, respectively. Scaling was performed by multiplying the nodal values of the best atlas with the percentage differences in each direction between the subject-of-interest and the used atlas.

## Loading Conditions

The selected loading inputs for simplified gait conditions were the same as those in our previous study,^29^ and those can be briefly described as follows:

1. We obtained generic gait loading input from previous experimental studies,^25^,^54^ including flexion angle and reaction forces through the tibiofemoral joint.
2. Based on the subject-of-interest, experimentally observed joint reaction forces through the tibiofemoral joint were scaled according to the subject’s body weight (Fig. 1d), and 50% of those forces were applied to the medial compartment.^25^,^29^
3. The load transfer of the meniscus was subtracted from the total reaction forces through the compartment. Using this method, we were able to considerably reduce the computational burden. Moreover, in a previous study,^27^ we demonstrated that this assumption has a negligible effect, i.e., 2, 1 and 0.7%, on the simulated stress, contact pressure, and pore pressure values, respectively. Therefore, we first calculated the forces through the medial and lateral menisci for each template, after which we calculated the average meniscus support as a fraction of the total force through the tibiofemoral contact for the medial and lateral menisci. In our previous study,^29^ we compared the models, including the subject-specific meniscus support force and average meniscus support force. Interestingly, we acquired comparable results for both models.

## Material Model and FE Simulations

Cartilages were modelled as a fibril reinforced poroviscoelastic (FRPVE) material,^29^,^46^,^52^,^54^ with the depth-dependent Benninghoff-type arcade architecture of collagen fibrils with split-line patterns.^10^ This complex cartilage material can distinguish between the different tissue constituents (collagen, proteoglycan, and fluid) and capture the tension-compression nonlinearity of cartilage.^23^,^52^ Concisely, this material includes a porohyperelastic nonfibrillar phase and a viscoelastic fibrillar phase. The total stress (**σ**_t_) of the material includes the non-fibrillar matrix stress (**σ**_nf_), the fibril network stress (**σ**_f_), and the fluid pressure (*p*)

1 $$ \varvec{\sigma}_{t} =\varvec{\sigma}_{\text{nf}} +\varvec{\sigma}_{\text{f}} - p{\mathbf{I}}, $$

where **I** is the unit tensor. In the fibrillar component, the fibrils were defined as primary and secondary fibrils; the primary collagen fibrils were oriented according to split-line patterns and depth-dependent architecture,^8^,^10^ whereas the secondary fibrils were randomly oriented in 13 different orientations.^52^ The parameters of the model which are based on a previous experimental study are listed in Table 1.^16^

**Table 1:** Material parameters implemented for cartilage.

FRPVE material parameters	Femoral cartilage	Tibial cartilage
*E*_m_ [MPa]	0.215	0.106
*E*_0_ [MPa]	0.92	0.18
*E*_e_ [MPa]	150	23.06
*ν*_m_	0.15	0.15
*η* [MPa s]	1062	1062
*k* [10^−15^ m^4^/Ns]	6	18
*n*_f_	0.8–0.15h_z_	0.8–0.15h_z_

*E*_m_ = nonfibrillar matrix modulus, *E*_0_ = initial fibril network modulus, *E*_e_ = strain-dependent fibril network modulus, *ν*_m_ = Poisson’s ratio of the nonfibrillar matrix, *η* = viscoelastic damping coefficient of fibrils, *k* = permeability, *n*_f_ = fluid fraction, and *h*_z_ = normalized depth

FE model construction and simulations were performed using the Abaqus finite element package (v6.13-3, Dassault Systèmes, Providence, RI, USA) (Fig. 1e), and the FRPVE material properties were implemented using the UMAT subroutine.

### Statistical Analysis

To statistically compare the measured anatomical dimensions in the CT and MR images, all of the length measurements were conducted three times by four different raters. Thereafter, the MRI- and CT-based FE models were generated based on the anatomical dimensions measured by one of the raters (simulating three trials). We selected the maximum principal stress, maximum principal strain, minimum principal strain, fibril strain, and pore pressure to allow the quantitative comparisons between the FE models. We obtained averaged (over the contact area) and peak values of the aforementioned parameters over the medial tibial compartment on the cartilage-cartilage contact as a function of stance. These mechanical parameters were selected because they have been linked to the failure and degeneration of cartilage tissue.^12^,^51^

We compared the total joint space width of the femoral and tibial cartilage (JSW medial and JSW lateral); the maximum anterior-posterior distance of the ellipse-like medial and lateral condyles (AP medial and AP lateral); and the maximum medial-lateral length (ML) using the linear mixed model with SPSS Statistics (IBM SPSS Statistics, v25, IBM Corp., Armonk, NY). This statistical model considers the potential dependency of the measurements performed by the same rater. In the model, the subjects were set as a random effect, while the imaging modality (MRI and CT) and raters were set as fixed variables.

We used the intraclass correlation coefficient (ICC) to study the repeatability of the aforementioned parameters in the CT and MRI modalities. We used a two-way random effects model for absolute agreement in the intrarater reliability analyses.^22^ The ICC values were interpreted according to Koo and Li^22^ with the following cutoff points: <0.5 poor, 0.5–0.75 moderate, 0.75–0.9 good, and >0.90 excellent reliability. All of the reliability analyses were performed using SPSS Statistics (IBM SPSS Statistics, v25, IBM Corp., Armonk, NY).

We employed 1-D statistical parametric mapping (SPM)^36^ in order to conduct the pairwise (subject-wise) comparison for the averaged (over the contact area) and peak values of the maximum principal stress, maximum principal strain, minimum principal strain, fibril strain, and pore pressure between the CT- and MRI-based models during the stance phase of the gait. We used the SPM method because of its advantage in considering multiple comparisons on smooth and random 1-D trajectories, compared to traditional (“0-D”) methods such as the parametric t-test or nonparametric Wilcoxon signed-rank test. As the number of subjects was small, the nonparametric SPM method was used for comparing the results of the model outcomes. SPM was performed using MATLAB. The limit of statistical significance for all comparisons was set to *p* < 0.05.

## Results

### Length Parameters (Anatomical Dimensions of Femur)

The mean values of the maximum medial-lateral length (ML), the maximum anterior-posterior distance of the medial and lateral condyles (AP medial and AP lateral), and the maximum joint space width in the medial and lateral condyles (JSW medial and JSW lateral), measured by different raters, are listed in Table 2. There was no significant difference (*p* > 0.05) between the anatomical dimensions measured by different raters. In Table 3, the aforementioned values are listed for both imaging modalities (MRI and CT). In general, there were no statistically significant differences (*p* > 0.05) in the values of parameters measured from the CT and MR images (Table 3). The only exception was that the joint space width at the lateral tibiofemoral contact area was greater in the MR images (Table 3) (*p* = 0.01).

**Table 2:** Mean values of the maximum medial-lateral length (ML), maximum anterior-posterior distance of the medial and lateral condyles (AP), and maximum joint space width (JSW) in the medial and lateral condyles measured by different raters.

	Mean, rater 1	Mean, rater 2	Mean, rater 3	Mean, rater 4	*p*-value
ML [mm]	79.84 ± 4.26	80.25 ± 4.29	79.72 ± 4.01	80.34 ± 4.41	0.84
AP medial [mm]	52.95 ± 4.27	53.33 ± 4.36	53.46 ± 4.37	52.98 ± 4.25	0.91
AP lateral [mm]	61.66 ± 4.31	61.77 ± 4.89	61.93 ± 4.49	62.26 ± 4.63	0.91
JSW medial [mm]	3.82 ± 1.23	3.84 ± 1.16	3.86 ± 1.20	3.86 ± 1.13	0.99
JSW lateral [mm]	5.38 ± 0.72	5.38 ± 0.87	5.62 ± 0.80	5.25 ± 1.02	0.17

**Table 3:** Mean values of the maximum medial-lateral length (ML), maximum anterior-posterior distance of the medial and lateral condyles (AP), and maximum joint space width (JSW) in the medial and lateral condyles for both imaging modalities.

	Mean, MRI	Mean, CT	*p*-value
ML [mm]	80.42 ± 3.95	79.65 ± 4.46	0.19
AP medial [mm]	52.81 ± 4.31	53.56 ± 4.25	0.20
AP lateral [mm]	62.23 ± 4.53	61.58 ± 4.59	0.31
JSW medial [mm]	3.90 ± 1.28	3.79 ± 1.06	0.53
JSW lateral [mm]	5.56 ± 0.95	5.25 ± 0.74	0.01

Both CT and MRI showed good intrarater reliability for the measured parameters (Table 4). There were no systematic differences (*p* > 0.05) between the intraclass correlation coefficient (ICC) scores of the MRI and CT measurements (Table 4). CT performed slightly better than MRI regarding intrarater reliability. Overall, 90 % (18 out of 20) of length measurements from the CT images were obtained with good or excellent reliability (ICC > 0.75), while this value was 80% (16 out of 20) for the measurements from the MR images.

**Table 4:** The intraclass correlation coefficient (ICC) obtained for each rater and both imaging modalities.

	Rater 1	Rater 2	Rater 3	Rater 4
ML, MRI	0.986 (0.953–0.997)	0.992 (0.976–0.998)	0.947 (0.841–0.987)	0.972 (0.916–0.993)
ML, CT	0.981 (0.945–0.995)	0.954 (0.870–0.988)	0.967 (0.904–0.992)	0.818 (0.514–0.919)
AP medial, MRI	0.939 (0.829–0.985)	0.737 (0.414–0.925)	0.899 (0.635–0.976)	0.818 (0.643–0.951)
AP medial, CT	0.861 (0.426–0.969)	0.917 (0.770–0.979)	0.800 (0.548–0.934)	0.892 (0.688–0.973)
AP lateral, MRI	0.919 (0.771–0.979)	0.973 (0.922–0.993)	0.861 (0.337–0.970)	0.685 (0.285–0.871)
AP lateral, CT	0.870 (0.430–0.971)	0.946 (0.846–0.986)	0.963 (0.889–0.991)	0.975 (0.927–0.994)
JSW medial, MRI	0.943 (0.840–0.985)	0.959 (0.881–0.990)	0.620 (0.232–0.885)	0.818 (0.487–0.918)
JSW medial, CT	0.883 (0.691–0.969)	0.731 (0.401–0.924)	0.857 (0.640–0.962)	0.763 (0.538–0.931)
JSW lateral, MRI	0.803 (0.515–0.947)	0.970 (0.913–0.992)	0.726 (0.400–0.921)	0.666 (0.266–0.852)
JSW lateral, CT	0.736 (0.416–0.924)	0.974 (0.924–0.994)	0.840 (0.604–0.957)	0.851 (0.627–0.960)

### Biomechanical Responses

**Figure 2:**
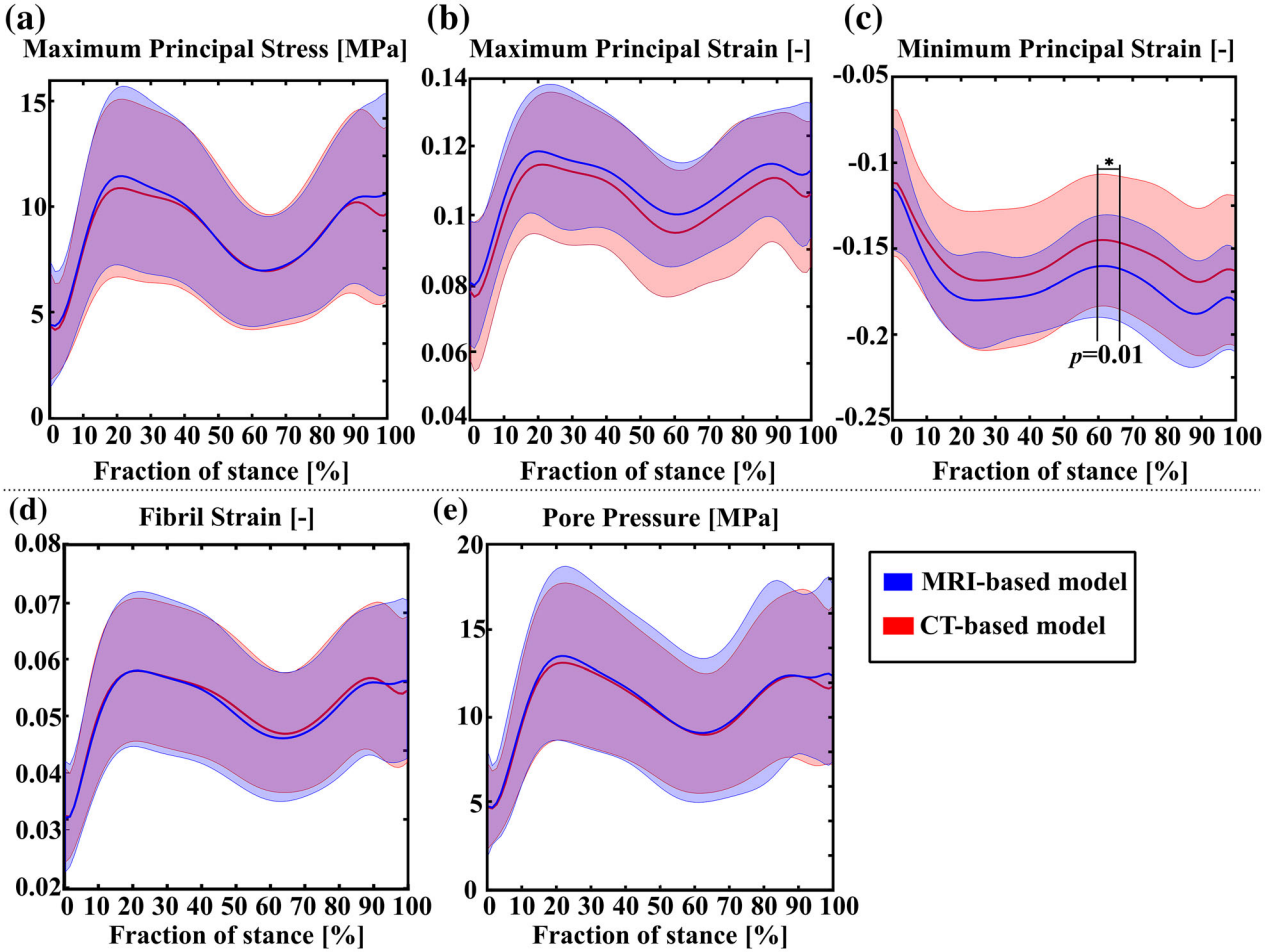
Comparison of the peak values for (a) maximum principal stress (tensile stress), (b) maximum principal strain (tensile strain), (c) minimum principal strain (compressive strain), (d) fibril strain, and (e) pore pressure (fluid pressure) between the MRI- and CT-based models during the stance phase of the gait. The solid line represents mean of the peak values, whereas the shaded area represents standard deviation.

Comparison of the peak values for maximum principal stress (tensile stress), maximum principal strain (tensile strain), minimum principal strain (compressive strain), collagen fibril strain, and pore pressure (fluid pressure) between the MRI- and CT-based models as a function of the stance is shown in Fig. 2. The only statistically significant difference between the CT- and MRI-based models was found in the minimum principal strain at around 60-65% of the stance (Fig. 2c). More detailed subject-specific comparisons for the peak values are presented separately for each parameter in the supplementary material (Figs. S1, S2, S3, S4 and S5).

**Figure 3:**
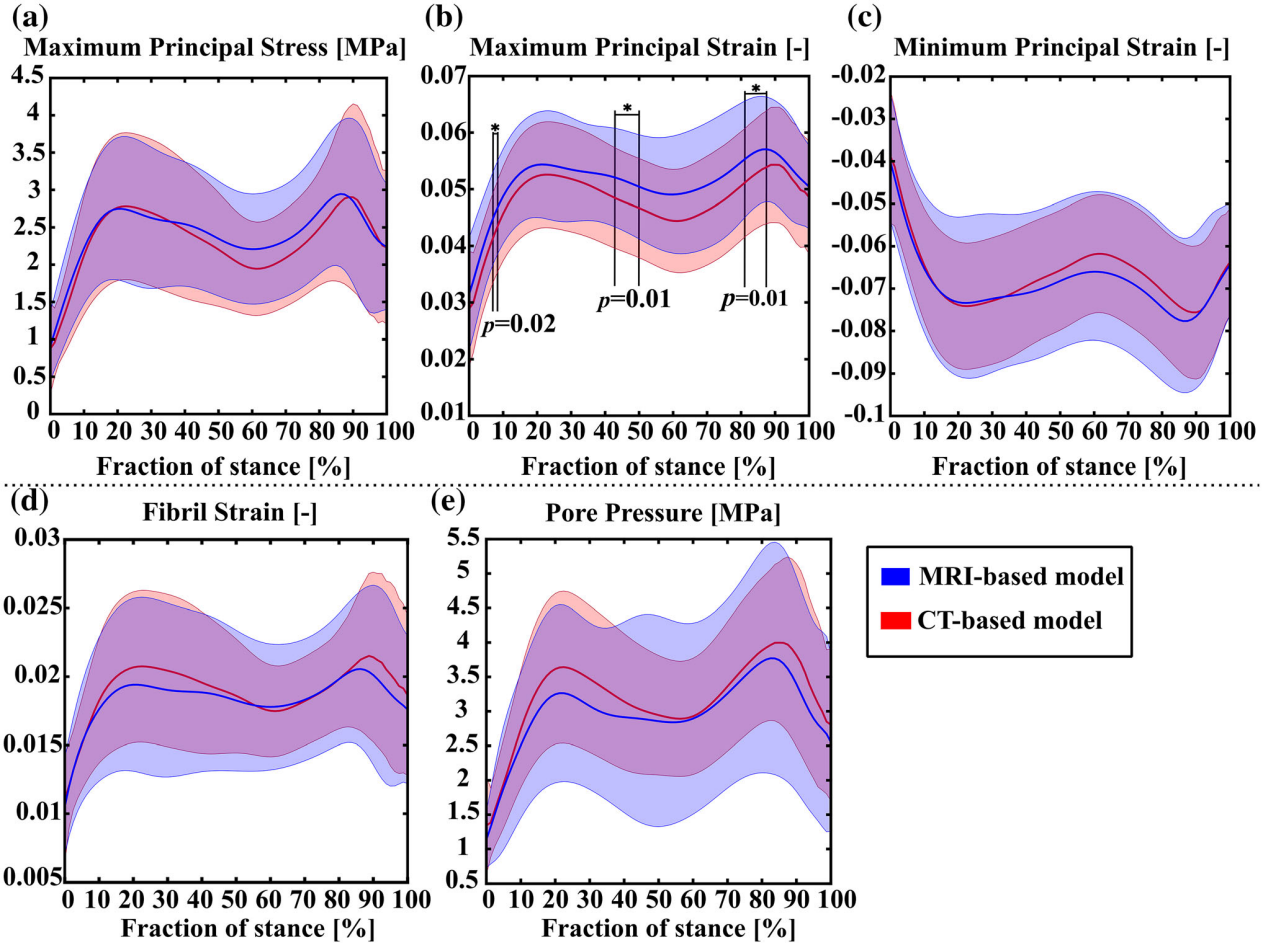
Comparison of the average values for (a) maximum principal stress (tensile stress), (b) maximum principal strain (tensile strain), (c) minimum principal strain (compressive strain), (d) fibril strain, and (e) pore pressure (fluid pressure) between the MRI- and CT-based models during the stance phase of the gait. The solid line represents mean of the peak values, whereas the shaded area represents standard deviation.

The mean values of the parameters averaged over the contact area during the stance phase are shown in Fig. 3. The only statistically significant differences between the CT- and MRI-based models were in the maximum principal strain at certain phases of the gait cycle (Fig. 3b).

## Discussion

We presented a rapid atlas-based framework for generating FE knee joint models with personalized cartilage volumes and topographies from contrast agent-free CT images to simulate biomechanical responses of cartilage. This approach aims at addressing the lack of rapid and reliable methods in FE modeling of the knee joint and contributes to filling the gap between clinical use and high-fidelity FE models, striking a compromise between accuracy, availability, manual effort and computational complexity. Our results illustrate the utility of our method, establishing its potential as a promising asset for evaluation of knee joint mechanics based on contrast agent-free CT images.

We evaluated the applicability of the presented framework by comparing the CT and MRI anatomical dimension measurements, and the results of FE models. Particularly, from five measured knee joint dimension parameters, there was only one parameter (the joint space width at the lateral tibiofemoral contact) that was statistically significantly different between the CT and MRI measurements. Moreover, 90% of measurements in CT and 80% of measurements in MRI had good or excellent reliability (ICC > 0.75). It should be noted that not all of the raters had previous experience in measuring joint anatomical dimensions from MR or CT images. Indeed, these promising results were acquired by two raters who had no previous experience with this method, a rater who had some previous experience and a rater who had developed the atlas-based method. The difference between the measured dimensions in the CT and MR images and the ICC scores for specific measurements may be due to the different spatial resolutions of the CT and MRI and/or minor differences in the knee alignment in different imaging modalities. The voxel size of the MR images used in this study was 0.6 × 0.6 × 0.6 mm^3^ (approximately 10–20% of the joint space), and for the CT images, the voxel size was 0.3 × 0.3 × 0.3 mm^3^ (approximately 5–10% of the joint space). Hence, a difference of even one-pixel in the measurements between the methods may cause differences in measured dimensions, especially when joint space widths are compared. Furthermore, in one of the subjects, femoral and tibial cartilages were not in contact with each other in the lateral compartment, which was confirmed by MRI. As in the CT-based modeling method, we assume that both medial and lateral compartments are in contact during clinical imaging, this may lead to overestimated cartilage thicknesses measured from CT images. This may also explain why there were differences between joint space width measured from CT and MR images.

In terms of biomechanical responses, there were no statistically significant differences in the peak and mean (over the contact area) values of most of the analyzed parameters between the CT- and MRI-based FE models. The only differences between the models were observed in the minimum and maximum principal strains in short periods of the gait. Moreover, for a few subjects, the results of the CT- and MRI-based models were not perfectly matched (supplementary material). These differences are presumably due to the anatomical dimension measurements and selection of templates. They may be minimized by improving the accuracy of knee dimension measurements, for instance, by employing (semi)automatic segmentation methods and by adding more knee templates to the atlas library.

In the literature, it has been suggested that excessive minimum principal strain, shear strain and deviatoric strain are associated with cell death and subsequent proteoglycan loss,^19^,^35^ while excessive collagen fibril strain and maximum principal stress have been associated with collagen failure, and initiation and progression of OA.^2^,^27^,^30^,^45^ The challenge is that it is not possible to define these properties in a subject-specific manner. It is only known that cartilage failure stress and strain decrease as a function of age.^20^ This property can be implemented in our model. Recently, using the atlas-based modeling method and 7 MPa threshold (modified by age, but not patient-specific) for the initial cartilage damage in a degeneration algorithm, we showed a good agreement between the predicted knee OA progression and the clinical 4-year follow-up data.^29^ That kind of approach may provide a clinical tool not only for the prediction but also for the simulation of the effect of different interventions on the OA progression (e.g. surgery, rehabilitation). Based on the result of the present study, we could apply that prediction approach now also for contrast agent-free CT images. However, here we only focused on comparing the simulated stresses and strains between the MRI- and CT-based approaches. Provided that subject-specific properties would change the values of the analyzed parameters, they would not change any of the conclusions drawn from the pairwise comparisons.

There are some limitations in this study. First, the presented framework does not include subject-specific motion data for FE simulations. For diagnostics of OA and joint disorders, imaging is typically used to aid decision making, whereas motion data is not feasible to be acquired in hospitals. The use of simplified gait loading can be justified. In the previous study, we investigated the effect of using simplified gait loading in the prediction of knee OA progression among clinically healthy subjects and compared the FE model predictions against experimental follow-up radiographic Kellgren-Lawrence (KL) grades.^29^ In that study, we showed that the atlas-based model with the simplified gait is able to predict knee OA based on the simulated volumetric cartilage degeneration. However, in the future, a similar atlas-based approach or a machine learning method can be added to our framework in order to generate more accurate and subject-specific motion data.

Second, the FE models used in this framework include only tibiofemoral cartilages. Adding the patellofemoral compartment as well as all ligaments, tendons and muscles considerably increases the computational burden, which is a drawback for the clinical application. To circumvent this, we included the effect of these structures, including ligament and muscle forces, in the total force applied to the joint.

Third, we modeled only the medial joint compartment. As mentioned in the original article,^29^ the reason for exploiting only the medial compartment is because knee OA is more frequent in the medial than lateral compartment.^7^ We are currently working on adding the lateral compartment in our workflow and testing the model with more experimental data.

Fourth, the presented approach can only be used for intact cartilages and does not account for defects or delaminated cartilage, which are known to be precursors for the initiation and development of OA.^3^,^24^ In the future, we aim to extend the presented workflow for subjects with cartilage defects and other joint injuries.

Fifth, we did not implement subject-specific material properties for each subject. Even though certain MRI sequences have been linked with the collagen network orientation and fixed charge density of cartilage,^39^–^41^ there are no *in vivo* imaging methods that can provide feasible estimations of the complex material parameters needed in our FE models. Thus, we used the same material properties for all subjects. Since the aim was to compare two imaging-based modeling methods, where the only difference was generation of the model geometry, implementing subject-specific material parameters in the models should not change the results.^29^

Finally, the only possible source for differences between the results was the measurement of anatomical dimensions, and subsequent template selection and generation of the model geometry. All the rest of the model inputs were the same in both approaches. Therefore, we conducted only repeatability tests for the measured anatomical dimensions and simulated sensitivity of the models to those measurements. However, in the future, the method can be expanded with comprehensive sensitivity studies to further clarify uncertainties associated with the model and ensure the reliability of the results.

In brief, this study demonstrates the potential of the atlas-based modeling approach for generating FE knee joint models from contrast agent-free CT images. The presented framework can provide an alternative pathway to apply computational modeling for evaluating knee joint mechanics, estimating possible failure locations in joints, and predicting knee OA progression.

## Electronic supplementary material

Below is the link to the electronic supplementary material.

Supplementary material 1 (DOCX 12808 kb)
