# Supplementary material for: Rapid CT-based Estimation of Articular Cartilage Biomechanics in the Knee Joint Without Cartilage Segmentation
Source: Ann Biomed Eng. 2020 Nov 11;48(12):2965–75. doi: 10.1007/s10439-020-02666-y (PMC7723937; doi:10.1007/s10439-020-02666-y)
Supplement: Supplementary file 1 — Supplementary material 1 (DOCX 12808 kb) [file 10439_2020_2666_MOESM1_ESM.docx]

**Supplementary material for:**

**Rapid CT-based Estimation of Articular Cartilage Biomechanics in the Knee Joint Without Cartilage Segmentation**

Ali Mohammadi^1^, Katariina A.H. Myller^2,3^, Petri Tanska^1^, Jukka Hirvasniemi^4^, Simo Saarakkala^5,6^, Juha Töyräs^1,2,7^, Rami K. Korhonen^1^, Mika E. Mononen^1^

^1^ Department of Applied Physics, University of Eastern Finland, Kuopio, Finland; ^2^Diagnostic Imaging Center, Kuopio University Hospital, Kuopio, Finland; ^3^Department of Medical Physics, Turku University Central Hospital, Turku, Finland; ^4^Department of Radiology & Nuclear Medicine, Erasmus University Medical Center, Rotterdam, the Netherlands; ^5^ Research Unit of Medical Imaging, Physics and Technology, Faculty of Medicine, University of Oulu, Oulu, Finland; ^6^Department of Diagnostic Radiology, Oulu University Hospital, Oulu, Finland; ^7^School of Information Technology and Electrical Engineering, The University of Queensland, Brisbane, Australia

***Corresponding Author:**

Ali Mohammadi, M.Sc.

Address: POB 1627, FI-70211, Kuopio, Finland

Telephone: +358 44 9694255

Email: [ali.mohammadi@uef.fi](mailto:ali.mohammadi@uef.fi) ; [ali.mohammadi.bioengineering@gmail.com](mailto:ali.mohammadi.bioengineering@gmail.com)

**Results**


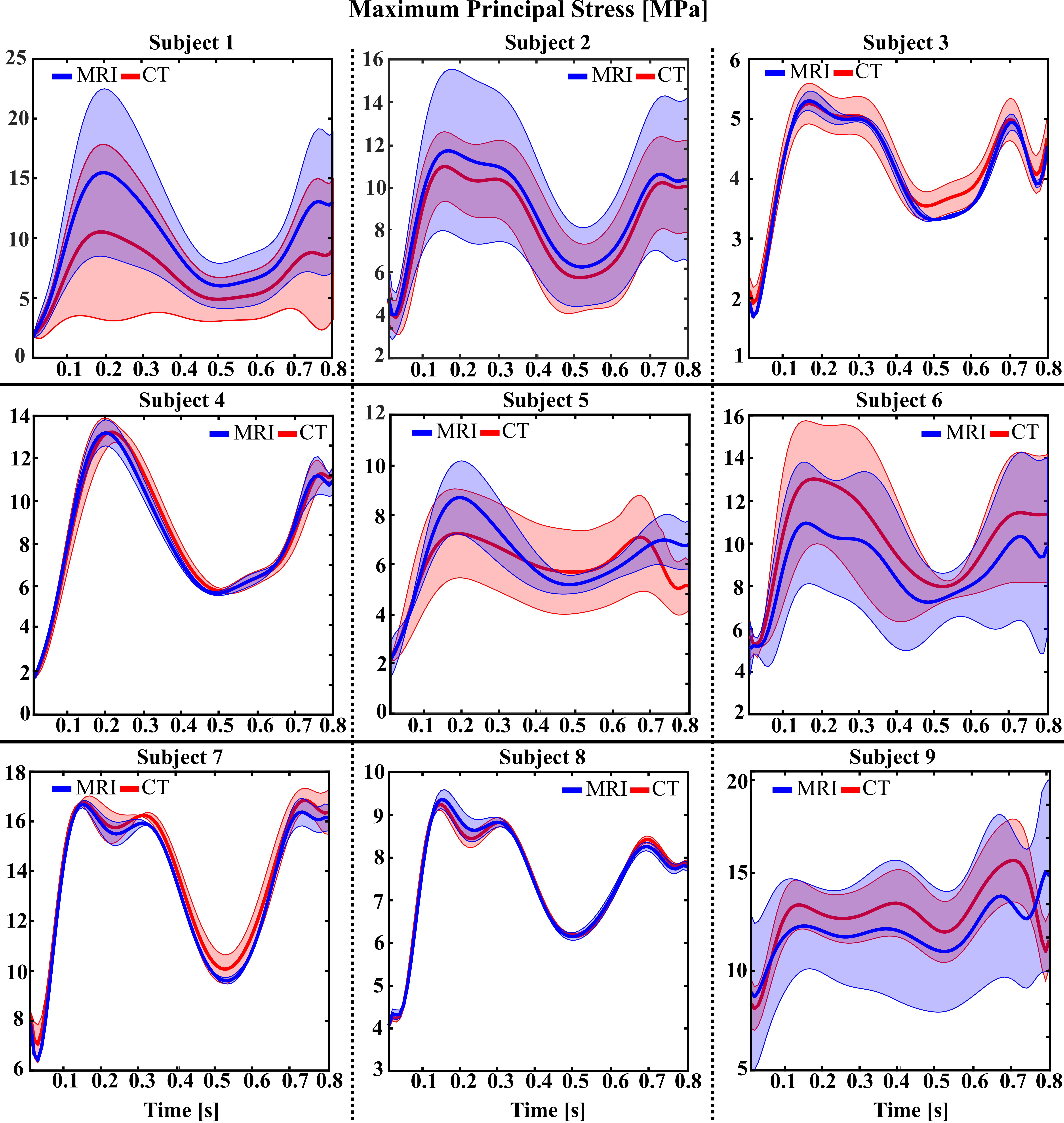
The subject-specific comparisons for the peak values of maximum principal stress, maximum principal strain, minimum principal strain, fibril strain and pore pressure are presented separately for each parameter in Figures S1, S2, S3, S4 and S5.

**Figure S1**: Peak values of the maximum principal stress at the contact area predicted by three CT- and MRI-based models for all nine subjects. The solid line represents the mean value from three trials, and the shaded area represents the standard deviation.


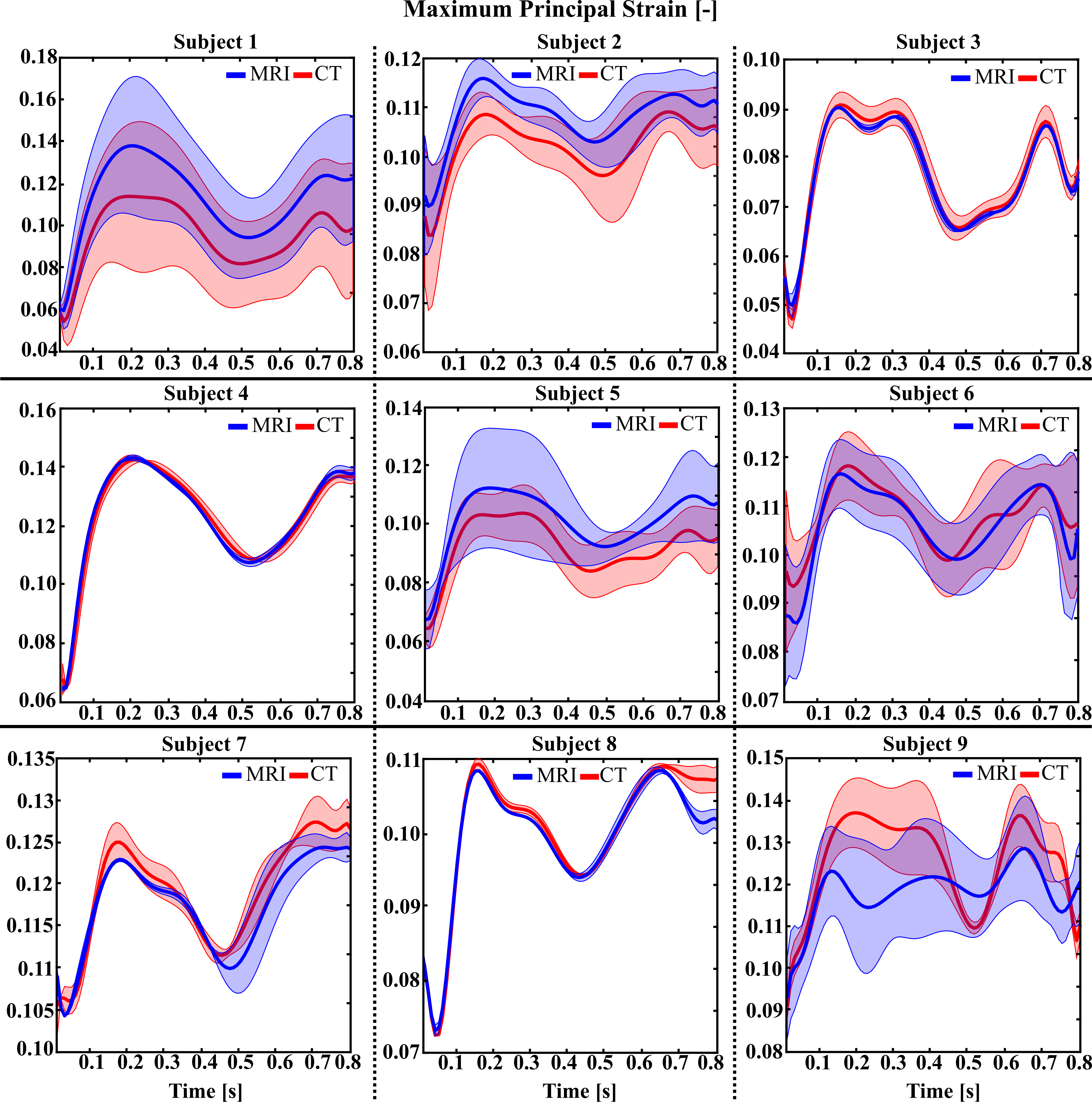


**Figure S2**: Peak values of the maximum principal strain at the contact area predicted by three CT- and MRI-based models for all nine subjects. The solid line represents the mean value from three trials, and the shaded area represents the standard deviation.


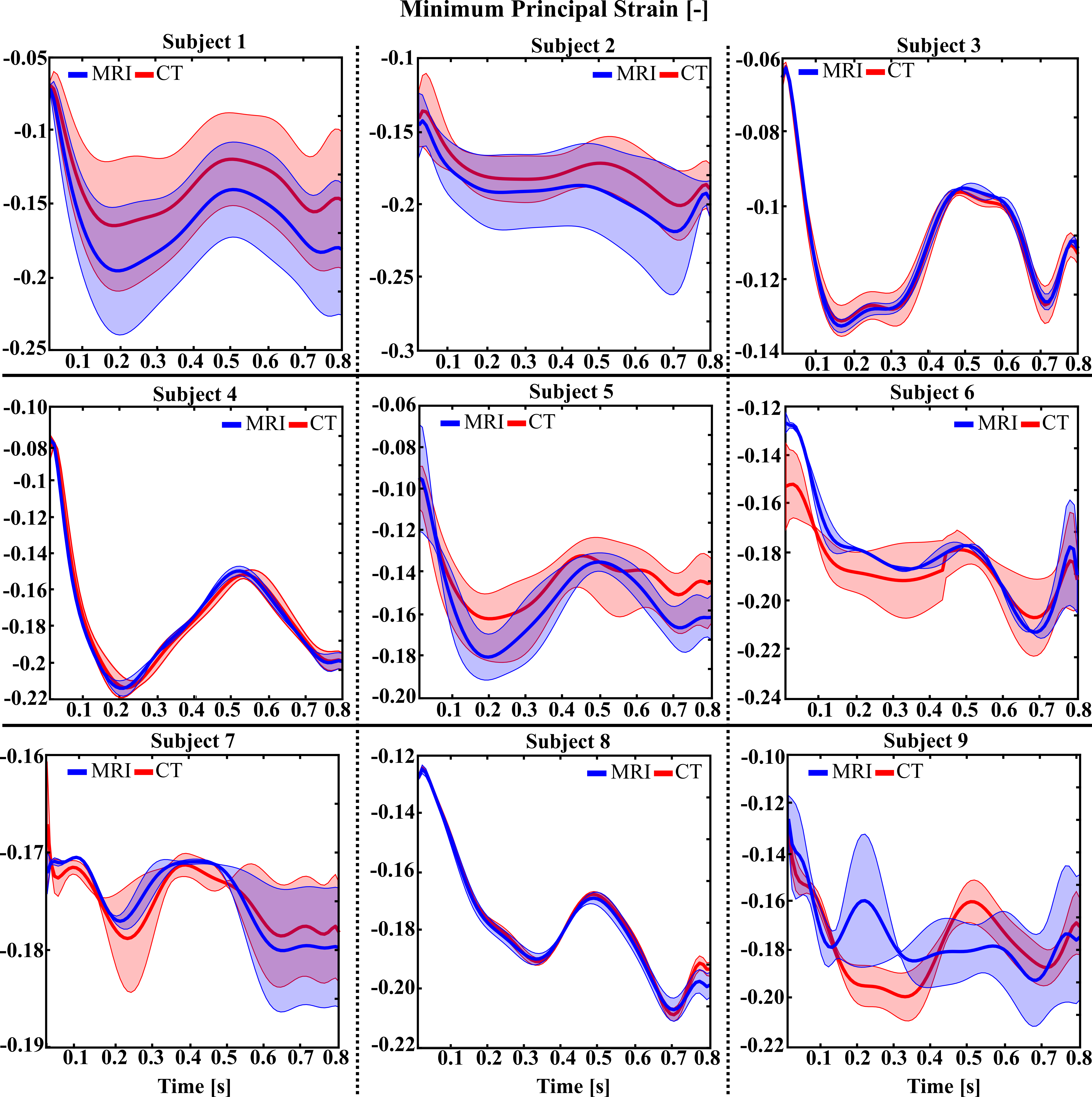


**Figure S3**: Peak values of the minimum principal strain at the contact area predicted by three CT- and MRI-based models for all nine subjects. The solid line represents the mean value from three trials, and the shaded area represents the standard deviation.


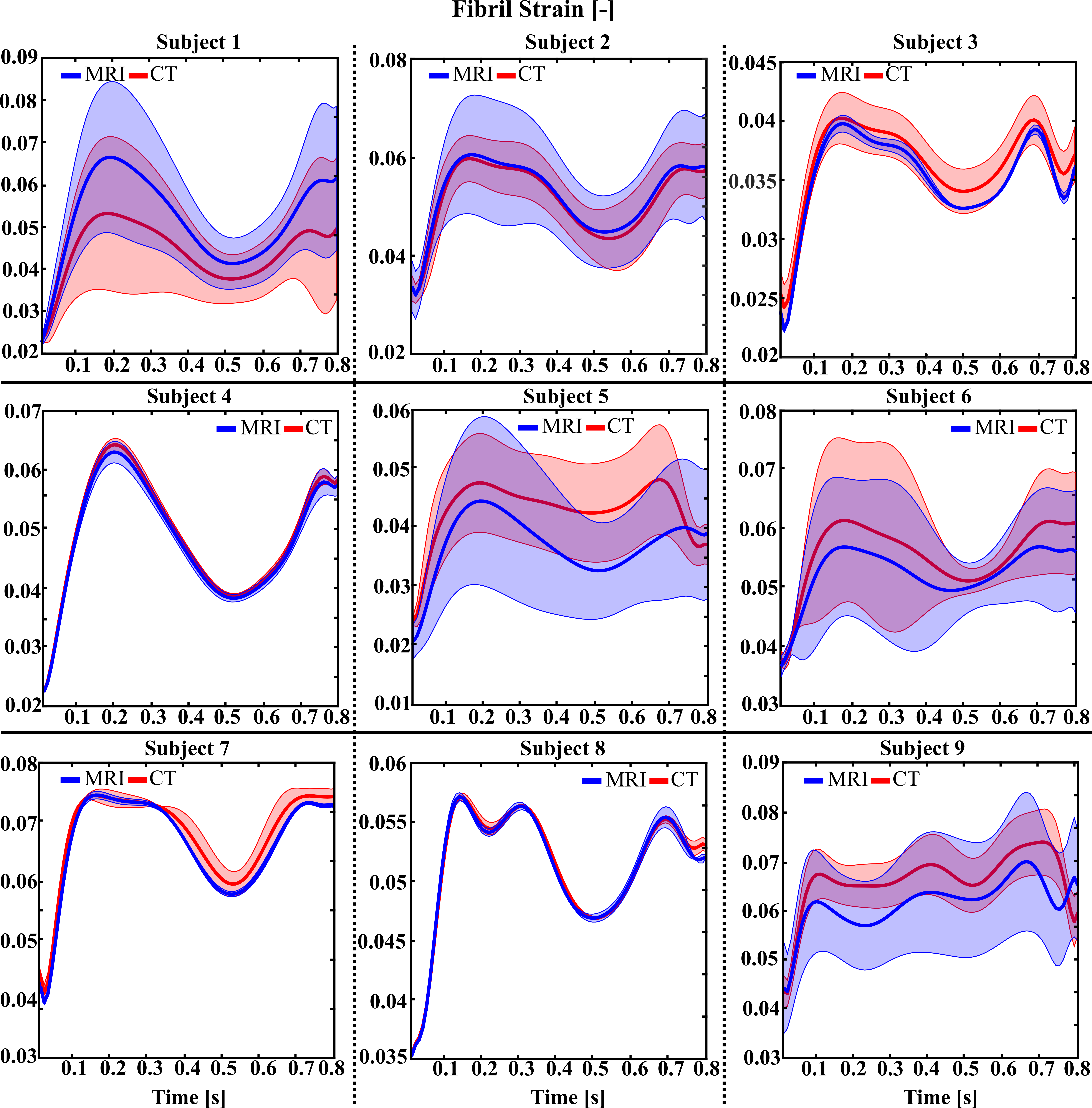


**Figure S4**: Peak values of the fibril strain at the contact area predicted by three CT- and MRI-based models for all nine subjects. The solid line represents the mean value from three trials, and the shaded area represents the standard deviation.


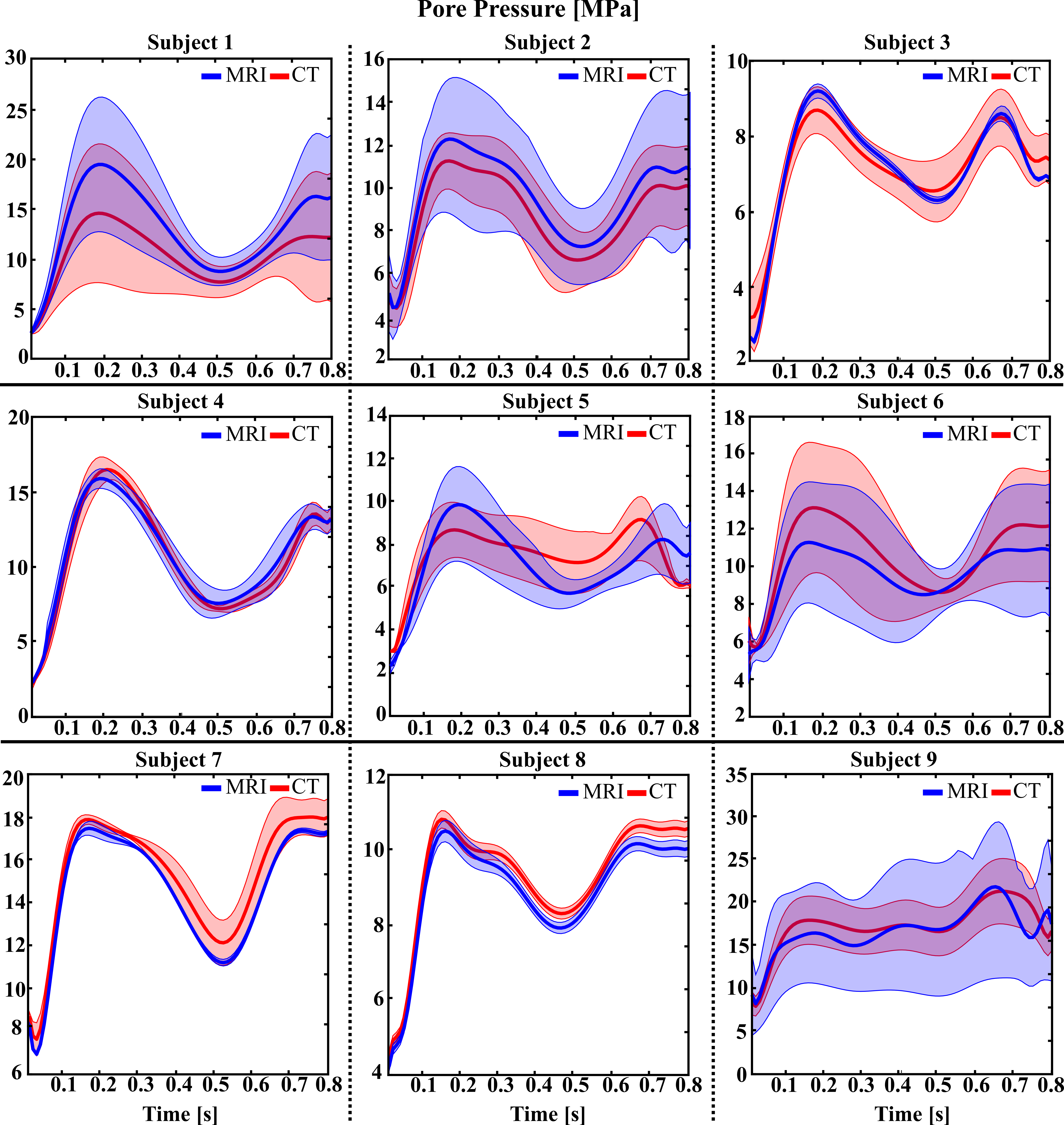


**Figure S5**: Peak values of the pore pressure at the contact area predicted by three CT- and MRI-based models for all nine subjects. The solid line represents the mean value from three trials, and the shaded area represents the standard deviation.
